# Touchscreen-based finger tapping: Repeatability and configuration effects on tapping performance

**DOI:** 10.1371/journal.pone.0260783

**Published:** 2021-12-07

**Authors:** Soma Makai-Bölöni, Eva Thijssen, Emilie M. J. van Brummelen, Geert J. Groeneveld, Robert J. Doll

**Affiliations:** 1 Centre for Human Drug Research, Leiden, the Netherlands; 2 Leiden University Medical Centre, Leiden, the Netherlands; University of Arkansas for Medical Sciences, UNITED STATES

## Abstract

Parkinson’s disease (PD) is a progressive neurodegenerative disease that affects almost 2% of the population above the age of 65. To better quantify the effects of new medications, fast and objective methods are needed. Touchscreen-based tapping tasks are simple yet effective tools for quantifying drug effects on PD-related motor symptoms, especially bradykinesia. However, there is no consensus on the optimal task set-up. The present study compares four tapping tasks in 14 healthy participants. In alternate finger tapping (AFT), tapping occurred with the index and middle finger with 2.5 cm between targets, whereas in alternate side tapping (AST) the index finger with 20 cm between targets was used. Both configurations were tested with or without the presence of a visual cue. Moreover, for each tapping task, within- and between-day repeatability and (potential) sensitivity of the calculated parameters were assessed. Visual cueing reduced tapping speed and rhythm, and improved accuracy. This effect was most pronounced for AST. On average, AST had a lower tapping speed with impaired accuracy and improved rhythm compared to AFT. Of all parameters, the total number of taps and mean spatial error had the highest repeatability and sensitivity. The findings suggest against the use of visual cueing because it is crucial that parameters can vary freely to accurately capture medication effects. The choice for AFT or AST depends on the research question, as these tasks assess different aspects of movement. These results encourage further validation of non-cued AFT and AST in PD patients.

## Introduction

Parkinson‘s disease (PD) is a progressive neurodegenerative disease that affects roughly 1 to 2% of the population above the age of 65 [1, 2]. The standard treatments remain symptomatic and novel treatments are continuously being investigated [3, 4]. One of the cardinal motor symptoms of PD is bradykinesia, defined as ‘slowness of voluntary movement initiation, progressive reduction of speed and amplitude of repetitive movement and difficulty of task switching’ [4]. Additional motor symptoms include tremor, muscular rigidity, and postural instability [4].

To assess the effectiveness of new (dopaminergic) medications, the Movement Disorder Society revised—Unified Parkinson’s Disease Rating Scale (MDS-UPDRS) serves as the ‘gold standard’ measurement [5]. This scale provides a wide range of assessments related to both motor and non-motor symptoms. Part III of the scale assesses motor symptoms, and its administration lasts approximately 15 minutes. However, the clinical rating scale is subject to varying inter-rater reliability, requires training and certification of the assessor, and is time-consuming for both the clinician and patient [6–9]. This may hamper the continuous assessment of (motor) symptoms, especially of rapid-acting agents. For instance, it will be difficult to accurately model the pharmacokinetic-pharmacodynamic (PK-PD) relationship of a medication with an early T_max_ (e.g., of less than 15–30 minutes) when using the time-consuming MDS-UPDRS part III as a pharmacodynamic measure. Hence, there is a need for short, reliable, and objective motor symptom quantification methods that are easy to implement in clinical research.

The number and variety of technologies aimed at quantifying PD motor symptoms has increased over the last decade [8, 10]. Many focus on finger tapping motions to quantify aspects of tremor, dyskinesia, and bradykinesia [8]. When quantifying bradykinesia, examples of technologies used vary from more rudimentary to increasingly sophisticated methods. For instance, arcade buttons [11], midi-keyboards [12], Inertial Measurement Units [13–19], and touchscreen devices [20–28] have all been used in previous studies. Touchscreen-based tapping tasks have been shown to not only differentiate reliably between PD patients and healthy controls (HCs) [12, 20–29] but also to detect medication effects [14, 20, 23, 29, 30]. Despite their potential in clinical research, there is no one standardized touchscreen based tapping task and seemingly minor configuration differences can affect the interpretation of study results [31].

Two variations of the touchscreen based finger tapping tasks are commonly described in literature: alternate tapping with the index and middle finger of one hand between two closely placed targets (Alternate Finger Tapping [AFT]) [22, 25, 32], and alternate tapping with the index finger between two targets placed on opposite ends of the screen (Alternate Side Tapping [AST]) [12, 20]. Each task assesses a different aspect of movement: whereas AFT requires fine finger movement, AST requires upper arm movement. Although studies report whether the AST and/or AFT was used, it is often unclear what the precise implementation of the tasks were (Table 1 for a brief overview of studies that used a finger tapping task). Varying target distances have been used both in AFT and AST. The inter target distance in AST studies varies between 1.5 cm to 25 cm. In studies using the AFT, most setups seem to place the targets under the natural position of the fingertips, yet, the precise inter-target distance is not always reported. Furthermore, both visually cued (e.g. [25] by changing target colors) and non-cued (e.g. [20] on a keyboard), versions of the test have been described. The distinction can be important as it has been shown that aiding PD patients with sensory cues can improve performance in finger tapping rhythm [33] and gait [34]. Most importantly, however, most studies are do not report all design choices, often omitting details about the inter-target distance, the presence or absence of a cue, or the task duration.

**Table 1:** Summary of the various finger tapping tasks found in the literature.

Study	Device	Target Distance (cm)	Cueing	Duration	Features
Alternate finger tapping tasks					
Arora [30, 32]	Phone application	N.A.	N.A.	N.A.	Numerous. E.g. speed, rhythm, accuracy, fatigue.
Lalvay [25]	Smartphone application (‘Mementum’)	N.A.	Alternating colors (red vs green)	20s	Regularity, rhythm, and changes in the number of taps
Tian [22]	Phone application	N.A.	N.A.	30s	Average number of buttons pressed between both hands
Alternate side tapping tasks					
Giancardo [27]	Arcade buttons	25	N.A.	Not clear (possibly 60s)	Average number taps between hands
Lipp [29]; Nutt [35]	Arcade buttons	20	N.A.	60s	Total number of taps
Hasan [20]	Keyboard	20	No	30s	Total number of taps, time spent on keyboards, rhythm, and dysmetria score
	iPhone application (‘TapPD’)	N.A.	Not clear:	30s	
			Changing colors		
	Tablet (‘TapPD’)	N.A.	Not clear:	30s	
			Changing colors		
Arroyo-Gallego [26]	Keyboard	25	N.A.	N.A	Not clear (possibly the total number of taps)
Mitsi [24]; Wissel [23]	Phone app	N.A.	N.A.	30s	Total number of taps, tap interval, tap duration, and tap accuracy
Young-Lee [21]	Tablet	1.5	N.A.	10s	Numerous. E.g. inter-tap distance, inter-tap interval time, total distance of a finger movement, and tapping speed.
Memedi [28]	Touch-pad with a pointer	2.7	N.A.	Not clear	Numerous. E.g. speed, accuracy, rhythm, and fatigue
			(Different target colors)	(possibly 20s)	

Abbreviations: AFT = alternate finger tapping, AST = alternate side tapping, N.A. = not available

To the best of our knowledge, no study has assessed the effects of cueing and task configuration in a comparative manner in healthy participants. The present study aims to compare four tapping tasks (cued/ non-cued AFT and AST) in healthy participants to identify the optimal design choices to be further validated in PD patients. First, the within- and between-day repeatability and (potential) sensitivity of the parameters are evaluated. Subsequently, the effect of the different configurations and cueing on tapping parameters are assessed.

## Methods

### Participants

No formal sample size calculations were performed since this was an exploratory, technical validation study. A total of 16 healthy participants were planned for enrolment. The number of participants was chosen to be of similar size as an early phase clinical trial and to achieve a balanced design. Inclusion criteria were self-reported normal or corrected vision and no self-reported significant health problems. Exclusion criteria included the presence of self-reported physical hand/arm impairment, any movement disorder (e.g., PD, essential tremor, dystonia, akinesia) and/or any other neurological condition. Participants were instructed to abstain from caffeine, smoking, and intensive physical exercise starting 12 hours prior to the tasks until the last measurement was completed. Participants gave consent prior to participation and did not receive any form of compensation. All data was collected anonymously (i.e., only age, gender, and handedness were collected). All procedures were approved by the internal Research Committee. The Research Committee considered the study a technical validation study that does not fall under the Dutch Medical Research Involving Human Subjects Act (WMO). Therefore, medical ethical approval from an independent medical-ethics committee was not required.

### Study design

**Fig 1:**
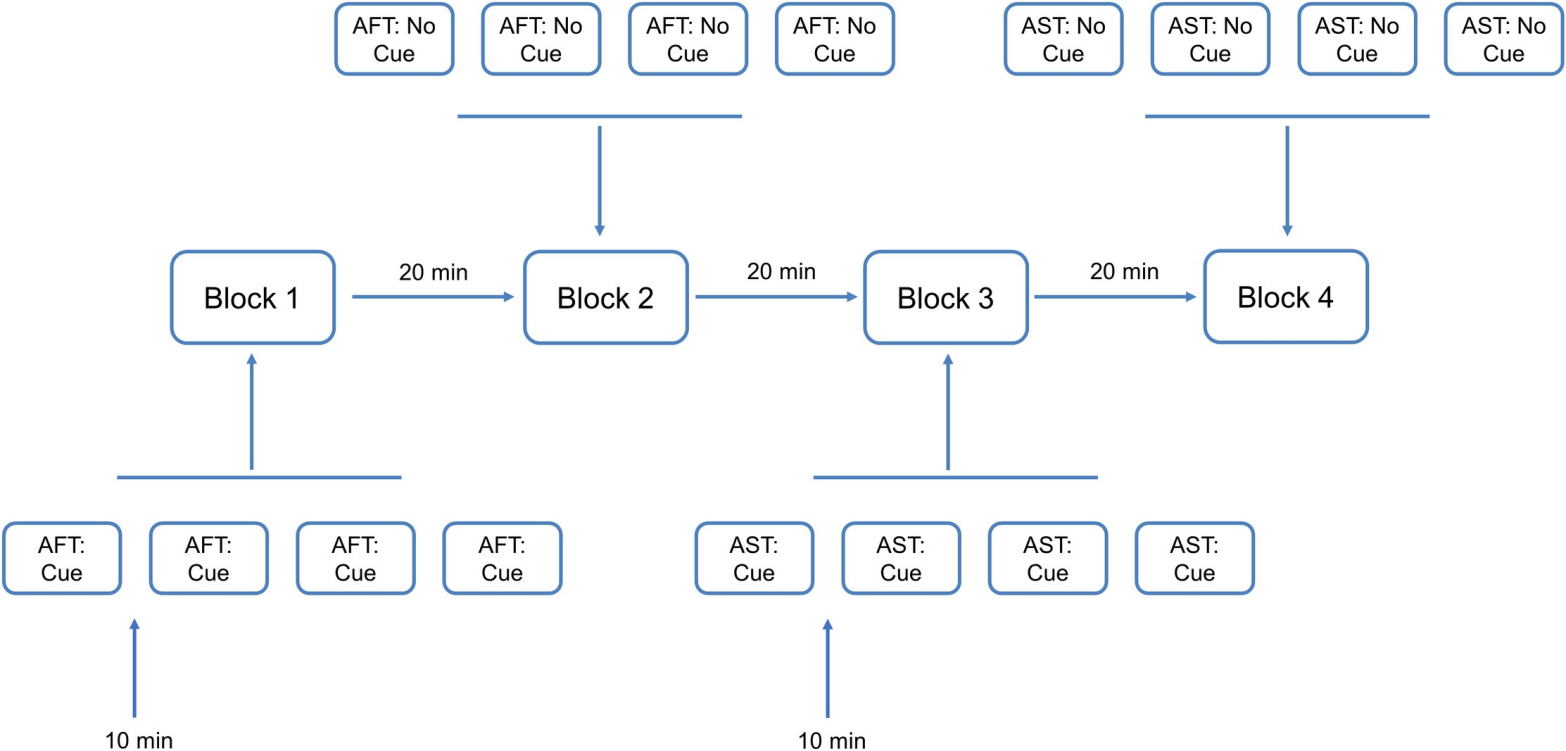
Timing and sequence of tapping tasks during both visits. The order of the experiments was counterbalanced using the Latin square method.

All participants visited the Centre for Human Drug Research (CHDR), Leiden, the Netherlands, twice, with a week between visits. To achieve a balanced design, the order of the blocks was counterbalanced using a Latin square method. Tapping tasks were conducted in the morning and their order was identical on both visits. Each task was performed four consecutive times, with 10-minute breaks between sessions. Participants were given a 20-minute break between two tapping tasks (for a schematic overview, see Fig 1). One visit lasted approximately 4 hours.

### Finger tapping tasks

**Fig 2:**
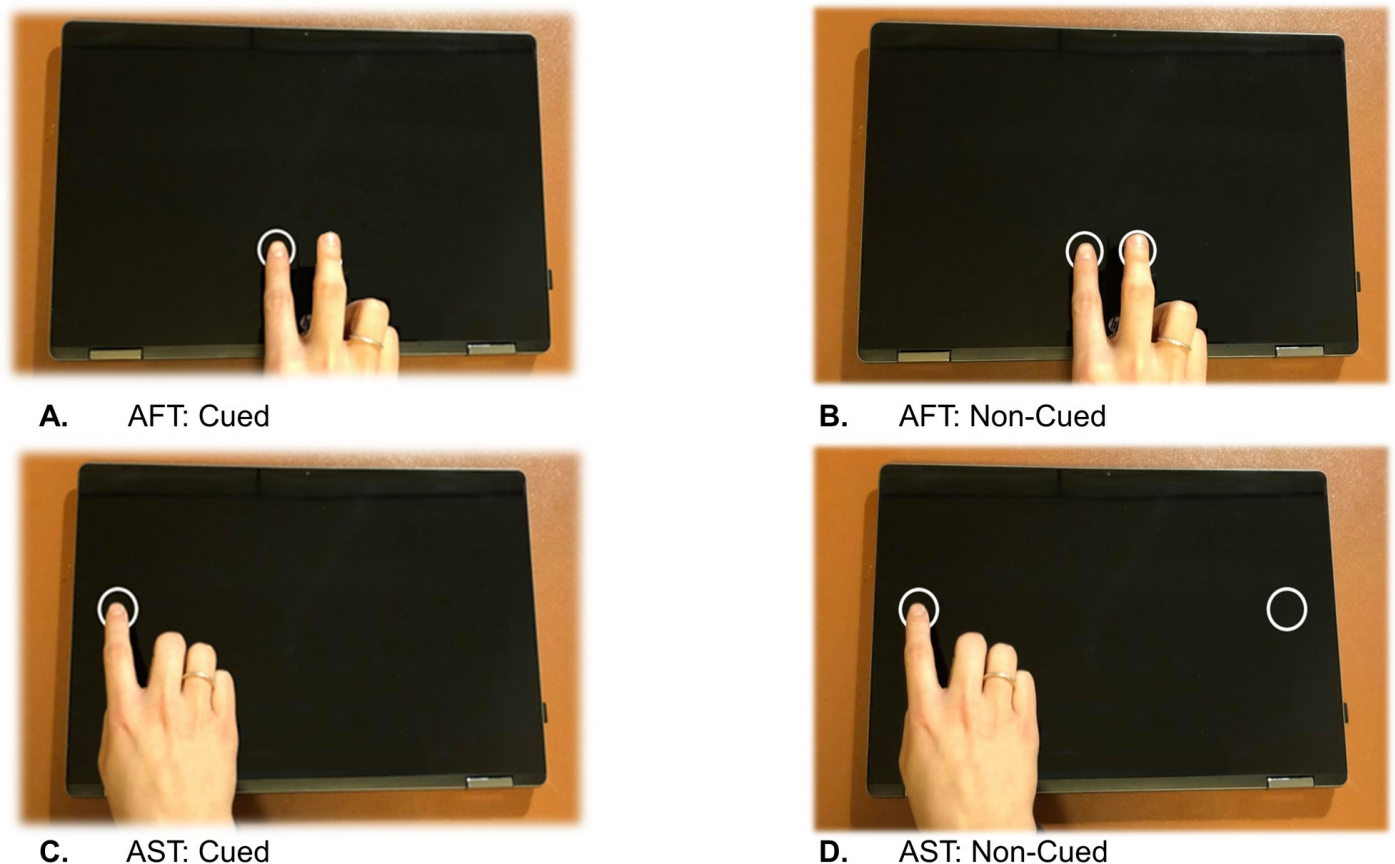
Finger tapping tasks. Figs A and B represent alternate finger tapping configuration (AFT). Figs C and D represent alternate side tapping (AST). In the cued configurations (A & C), the second circle only appears when a tap inside the target was successfully performed. B & D represent the non-cued tapping tasks.

All finger tapping tasks were performed with a touchscreen laptop (HP Pavilion x360; resolution = 1920 x 1080 pixels; screen width = 31 cm; screen height = 17.4 cm). The tasks were developed in-house using the Python programming language (version 3.4 [36]). The PsychoPy [37] library was used for stimulus presentation. The visual stimuli were two white circles (radius = 1 cm) placed horizontally on the screen on a black background. The two circles were either 2.5 or 20 cm apart, corresponding to the AFT and AST task, respectively. Depending on the configuration, targets were presented with or without a visual cue. With visual cueing, one target is visible at a time and only when tapped correctly does this target disappear while the other appears. Hence, a total of four tapping tasks were tested: cued and non-cued AFT, as well as cued and non-cued AST (see Fig 2).

**Fig 3:**
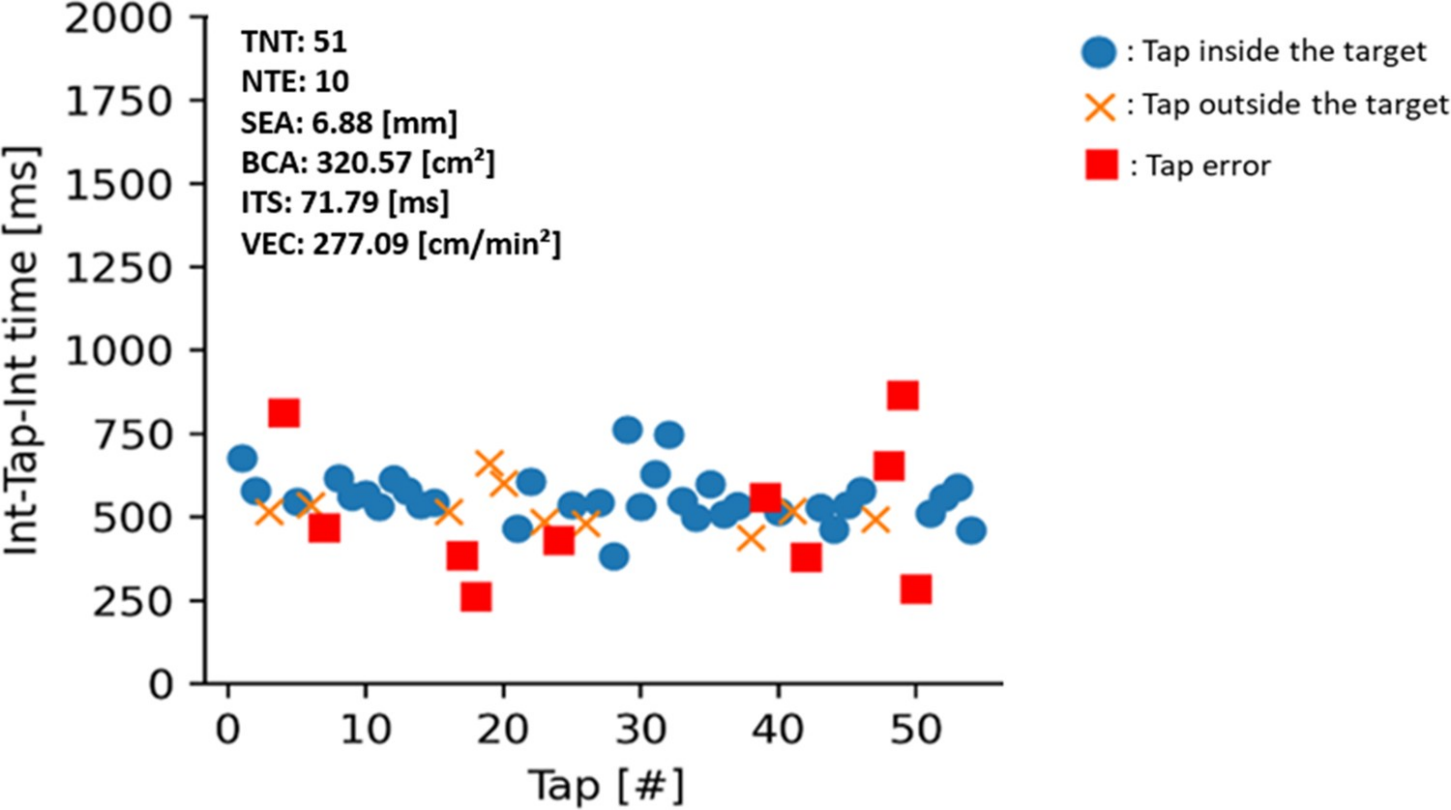
Data output example. Abbreviations: TNT = Total Number of Taps; NTE = Number of Tapping Errors; SEA = Spatial Error: Mean; BCA = Bivariate Contour Ellipse Area; ITS = Inter Tap Interval: Standard Deviation; VEC = Velocity: Change.

Tapping position (X and Y coordinates) and tapping time for each tap were registered. Parameters related to speed, accuracy, fatigue and rhythm were quantified for each of the four tapping tasks [28]. We calculated the total number of taps (TNT) as a proxy for tapping speed; the number of tapping errors (NTE), mean spatial error (SEA), and bivariate contour ellipse area (BCA), as variables of accuracy; the inter-tap interval standard deviation (ITS) representing rhythm; and the change in velocity (VEC) to capture fatigue (see Table 2 for an overview of the tapping task parameters and Fig 3 for a visual representation of the data output).

**Table 2:** Tapping task parameters.

Category	Parameter		Definition
**Speed**	Total Number of Taps (#)	TNT	Sum of all taps on the screen
**Accuracy**	Number of Tapping Errors (#)	NTE	The number of two (or more) consecutive taps on the same target;
	Spatial Error: Mean (mm)	SEA	Average absolute Euclidean distance from the target’s center point
	Bivariate Contour Ellipse Area (mm^2^)	BCA	Based on Castet & Crossland [38]:
			A bivariate contour ellipse encompassing a proportion of the highest density of finger taps:
			*BCA* = 2*χ*^2^*πσ_H_σ_V_*(1−*ρ*^2^)
			where, *χ*^2^ is a chi-square variable with 2 degrees of freedom;
			*σ_H_* and *σ_V_* is the SD of the horizontal (X) and vertical (Y) coordinates, respectively;
			*ρ* is the product-moment correlation of the two position components
**Rhythm**	Inter-Tap Interval: SD (ms)	ITS	The SD of the time between two consecutive taps
**Fatigue**	Velocity: Change (cm/min^2^)	VEC	A linear slope fitted on all inter-tap velocity values. Velocity was calculated by dividing the inter-tap distance value by the inter-tap interval

Abbreviation: SD = standard deviation

During all tapping tasks, participants were instructed to tap as accurately and fast as possible for 30 seconds. Participants used the index finger of their dominant hand during the AST tasks, whereas they used the index and middle finger alternately during the AFT tasks. Additionally, during the AST tasks, participants were asked to keep their elbow fixed in place on the table to prevent additional movement compensation.

### Statistical analysis

All data processing was performed via custom scripts in Python (version 3.8; [36]). Statistical modeling was performed using custom scripts as well as the ‘lme4’ [39] and ‘emmeans’ packages [40] in the R software package [41].

### Repeatability

To assess the repeatability of the parameters, the available dataset was split into two subsets to separately assess the within- and between-day repeatability. For within-day repeatability, only measurements from the first visit were considered. For between-day repeatability, data from both visits was used, but from each visit the four measurements were averaged.

For each parameter and subset, a random intercept Linear Mixed Model (LMM) was fit. For within-day repeatability, both the intercept and measurement number (i.e., 1 to 4) were included as fixed effects. For between-day repeatability, both the intercept and visit number (i.e., 1 and 2) were included as fixed effects. Based on the models, the intra-class correlation (ICC) was calculated by dividing the between-subject variance by the total variance (i.e., the sum of the between-subject variance and the within-subject error variance) [42]. Excellent degree of repeatability was considered for ICC values above .90, good for ICC values between .75 –.90, moderate for ICC values between .50 - .75, and poor for ICC values below .50 [42].

### Minimum detectable effect

To assess potential sensitivity, minimum detectable effect (MDE) values were calculated. First, a random intercept model including measurement number (i.e., 1 to 4) as fixed effect was fitted for each parameter. For each fitted model, fixed intercept, random intercept variance and residual variance were extracted. The MDE was then calculated by multiplying the effect size by the pooled standard deviation (i.e., the square root of the sum of the within- and between-subject variance) and expressed in terms of percentage change relative to the intercept value. The effect size used to calculate the MDE was based on a paired sample t-test with a power of .80, a significance level of 5% (α = .05), and a sample size of 20 (a typical sample size for a clinical).

### Effect of task configuration on performance

To assess the effect of configuration, cueing, measurement number, and visit number, a LMM was fitted for each parameter. For each model, the intercept, configuration (i.e., AFT or AST), cueing (i.e., cued or non-cued), measurement number (i.e., 1 to 4), and visit (i.e., 1 or 2) were included as fixed effects. Additionally, a two-way interaction between cueing and configuration was included as fixed effect. Between-subject random effects were included for the intercept. A more elaborate random structure was not possible without running into convergence issues. Type-III F-statistics were used to assess statistical significance of the fixed effects (α = .05). Where the interaction effect between the fixed effects was found to be significant, post-hoc pairwise comparisons with Tukey p-value correction were evaluated using the ‘emmeans’ package. Degrees of freedom for F-statistic denominators as well as pairwise comparisons were estimated via the Kenward-Roger method [43]. For pairwise comparisons, the effect size was estimated by calculating Cohen’s *d*. Effect sizes were considered small, medium, or large for values of d smaller than .20, between .20 and .50, or larger than .80, respectively [44].

## Results

Two participants could not be measured due to emerging COVID restrictions, hence data from 14 participants was collected (mean age: 25.6 ± SD: 3.1; 6 females, 13 right-handed). All but one of the participants successfully completed all measurements. For one participant, the first four measurements were not performed due to technical difficulties. A total of 444 tapping experiments were performed, resulting in 61103 recorded taps.

### Repeatability

The within-day repeatability of the six parameters in cued/ non-cued AFT and AST tasks are presented in Table 3. Excellent to good repeatability was observed in the speed parameter (i.e., total number of taps) across all tasks (ICCs > .86). The number of tapping errors showed good to moderate repeatability in AFT (ICC_cued_ = .81, ICC_non-cued_ = .69), but poor repeatability in AST (ICC_cued_ = .41, ICC_non-cued_ = .08). The mean spatial error showed good repeatability in AFT (ICC_cued_ = .79, ICC_non-cued_ = .75), and good to moderate repeatability in AST (ICC_cued_ = .67, ICC_non-cued_ = .84). Good to poor repeatability was observed in the bivariate contour ellipse area in AFT (ICC_cued_ = .77, ICC_non-cued_ = .05), and good to moderate repeatability in AST (ICC_cued_ = .67, ICC_non-cued_ = .84). The rhythm parameter, inter-tap interval SD, showed good repeatability in both AFT tasks (ICC_cued_ = .86, ICC_non-cued_ = .84), while it showed moderate to poor repeatability in AST (ICC_cued_ = .20, ICC_non-cued_ = .51). The change in velocity parameter showed moderate repeatability in AFT (ICC_cued_ = .56, ICC_non-cued_ = .58) and moderate to poor in AST (ICC_cued_ = .25, ICC_non-cued_ = .55).

**Table 3:** Within-day repeatability.

		Finger Tap	Side Tap
Feature		ICC [95% CI]	ICC [95% CI]
**TNT [#]**	Cued	.94 [.89, .97]	.86 [.76, .94]
	Non-cued	.90 [.82, .96]	.94 [.89, .98]
**NTE [#]**	Cued	.81 [.67, .91]	.41 [0.19, .66]
	Non-cued	.69 [.5, .86]	.08 [-.08, .37]
**SEA [mm]**	Cued	.79 [.64, .90]	.63 [.43, .82]
	Non-cued	.75 [.57, .88]	.76 [.60, .89]
**BCA [mm** ^**2**^ **]**	Cued	.77 [.61, .89]	.67 [.47, .83]
	Non-cued	.05 [-.12, .32]	.84 [.71, .92]
**ITS [ms]**	Cued	.86 [.76, .94]	.20 [.00, .48]
	Non-cued	.84 [.72, .93]	.51 [.30, .74]
**VEC [cm/min** ^**2**^ **]**	Cued	.56 [.34, .77]	.25 [.04, .53]
	Non-cued	.58 [.34, .78]	.55 [.34, .77]

Abbreviations: TNT = Total Number of Taps; NTE = Number of Tapping Errors; SEA = Spatial Error: Mean; BCA = Bivariate Contour Ellipse Area; ITS = Inter Tap Interval: Standard Deviation; VEC = Velocity: Change; ICC = Intra-Class Correlation, CI = Confidence Interval.

The between-day repeatability values for the six parameters are presented in Table 4. An excellent to good repeatability was observed in the total number of taps across all tapping tasks (ICCs: .78 –.97). The number of tapping errors showed excellent to good repeatability in AFT (ICC_cued_ = .96, ICC_non-cued_ = .81) and moderate to poor repeatability in AST (ICC_cued_ = .54, ICC_non-cued_ = .06). Of the accuracy parameters, mean spatial error showed moderate to good repeatability in AFT (ICC_cued_ = .80, ICC_non-cued_ = .70), and moderate in AST (ICC_cued_ = .53, ICC_non-cued_ = .56). The bivariate contour ellipse area showed moderate to poor repeatability in AFT (ICC_cued_ = .60, ICC_non-cued_ = .29), and moderate in AST (ICC_cued_ = .73, ICC_non-cued_ = .63). The rhythm parameter, inter-tap interval SD, showed good to moderate repeatability in AFT (ICC_cued_ = .85, ICC_non-cued_ = .52), and good to poor repeatability in AST (ICC_cued_ = .40, ICC_non-cued_ = .75). The change in velocity showed good to moderate repeatability in AFT (ICC_cued_ = .79, ICC_non-cued_ = .66) and good repeatability in non-cued AST (ICC_non-cued_ = .85). For cued AST, an ICC could not be estimated due to the model not converging.

**Table 4:** Between-day repeatability.

		AFT	AST
Feature		ICC [95% CI]	ICC [95% CI]
**TNT [#]**	Cued	.97 [.93, .99]	.78 [.51, .91]
	Non-cued	.86 [.68, .94]	.88 [.71, .95]
**NTE [#]**	Cued	.96 [.89, .98]	.54 [.13, .79]
	Non-cued	.81 [.58, .92]	.06 [-.39, .49]
**SEA [mm]**	Cued	.80 [.55, .92]	.53 [.11, .78]
	Non-cued	.70 [.38, .87]	.56 [.15, .80]
**BCA [mm** ^**2**^ **]**	Cued	.60 [.21, .82]	.73 [.43, .89]
	Non-cued	.29 [-.17, .65]	.63 [.26, .84]
**ITS [ms]**	Cued	.85 [.65, .94]	.40 [-.06, .71]
	Non-cued	.52 [.01, .78]	.75 [.47, .90]
**VEC [cm/min** ^**2**^ **]**	Cued	.79 [.53, .91]	-
	Non-cued	.66 [.30, .85]	.85 [.65, .94]

Abbreviations: TNT = Total Number of Taps; NTE = Number of Tapping Errors; SEA = Spatial Error: Mean; BCA = Bivariate Contour Ellipse Area; ITS = Inter Tap Interval: Standard Deviation; VEC = Velocity: Change; ICC = Intra-Class Correlation; CI = Confidence Interval;—: value could not be estimated due to the model not converging

### Minimum detectable effect

The calculated MDE values, expressed in percentages as well as in absolute values, can be found in Table 5. Generally, the MDE values for the AST configuration were lower than for AFT. The parameters having the lowest MDE values were the total number of taps, the mean spatial error, and the rhythm parameter (MDE values ranging from 9.5%– 23% in AST, and 19%– 71% in AFT).

**Table 5:** Sensitivity (MDE) estimates in percentage (%) and absolute values (Abs).

		AFT	AST
Feature		*MDE* [Abs]	*MDE* [Abs]
**TNT [#]**	Cued	28% [45]	9.5% [6.2]
	Non-cued	19% [37]	11% [9.5]
**NTE [#]**	Cued	98% [6.1]	57% [1.5]
	Non-cued	49% [6.7]	150% [0.54]
**SEA [mm]**	Cued	24% [0.73]	12% [0.54]
	Non-cued	20% [0.54]	12% [0.56]
**BCA [mm** ^**2**^ **]**	Cued	48% [22]	35% [55]
	Non-cued	88% [29]	26% [55]
**ITS [ms]**	Cued	32% [31]	23% [19]
	Non-cued	71% [68]	20% [8.4]
**VEC [cm/min** ^**2**^ **]**	Cued	-	90% [400]
	Non-cued	43% [–370]	170% [–460]

Abbreviations: MDE = Minimum detectable effect; Abs = Absolute value; TNT = Total Number of Taps; NTE = Number of Tapping Errors; SEA = Spatial Error: Mean; BCA = Bivariate Contour Ellipse Area; ITS = Inter Tap Interval: Standard Deviation; VEC = Velocity: Change;—: value could not be estimated due to the model not converging

### Effect of task configuration and cueing on tapping performance

The results of all LMM models are presented in Table 6. The configuration (i.e., AFT vs AST) had a significant effect on all parameters. Cueing affected all parameters except the mean spatial error. Lastly, a significant interaction effect between configuration and cueing was found for all parameters except the total number of taps and change in tapping velocity. None of the parameters were affected by the measurement number, see Table 6. However, the total number of taps, mean spatial error, and the inter-tap interval SD were affected by visit. For the pairwise comparisons between testing visits, see Table 7. On the second visit, participants tapped more often than on the first visit (*p* < .01). Moreover, the mean spatial error on the second visit was higher than on the first visit (*p* < .05). Finally, the inter-tap interval SD was lower on the second visit than on the first visit (*p <* .01).

**Table 6:** F-Test results of fixed effects for each parameter.

Category	Speed	Accuracy			Rhythm	Fatigue
**Parameter**	TNT	NTE	SEA	BCA	ITS	VEC
	*F*_(1, 423.05)_	*F*_(1, 423.07)_	*F*_(1, 423.07)_	*F*_(1, 423.08)_	*F*_(1, 423.14)_	*F*_(1, 423.16)_
**Configuration**	1412.11 ***	281.97 ***	593.15 ***	965.02 ***	80.14 ***	98.70 ***
**Cueing**	36.82 ***	5.61 *	0.01	4.77 *	5.87 *	37.03 ***
**Measurement**	0.95	0.83	0.13	0.76	0.47	0.21
**Visit**	10.61 **	0.30	7.08 **	0.72	8.51 **	0.67
**Configuration × Cueing**	0.33	37.24 ***	16.28 ***	10.15 **	12.78 ***	1.64

Abbreviations: TNT = Total Number of Taps; NTE = Number of Tapping Errors; SEA = Spatial Error: Mean; BCA = Bivariate Contour Ellipse Area; ITS = Inter Tap Interval: Standard Deviation; VEC = * Velocity: Change

* p < .05

** p < .01

*** p < .001

**Table 7:** Occasion effects on tapping performance.

Category	Speed	Accuracy			Rhythm	Fatigue
**Parameter**	TNT	NTE	SEA	BCA	ITS	VEC
**[unit]**	[#]	[#]	[mm]	[mm^2^]	[ms]	[cm/min^2^]
**visit 1- visit 2**	-9.86 **	0.29	-0.19 **	-3.99	12.1 **	-46.1
**(SE)**	(3.03)	(0.53)	(0.07)	(4.7)	(4.14)	(56.4)

Abbreviations: TNT = Total Number of Taps; NTE = Number of Tapping Errors; SEA = Spatial Error: Mean; BCA = Bivariate Contour Ellipse Area; ITS = Inter Tap Interval: Standard Deviation; VEC = Velocity: Change; SE = standard error

* p < .05

** p < .01

*** p < .001

**Fig 4:**
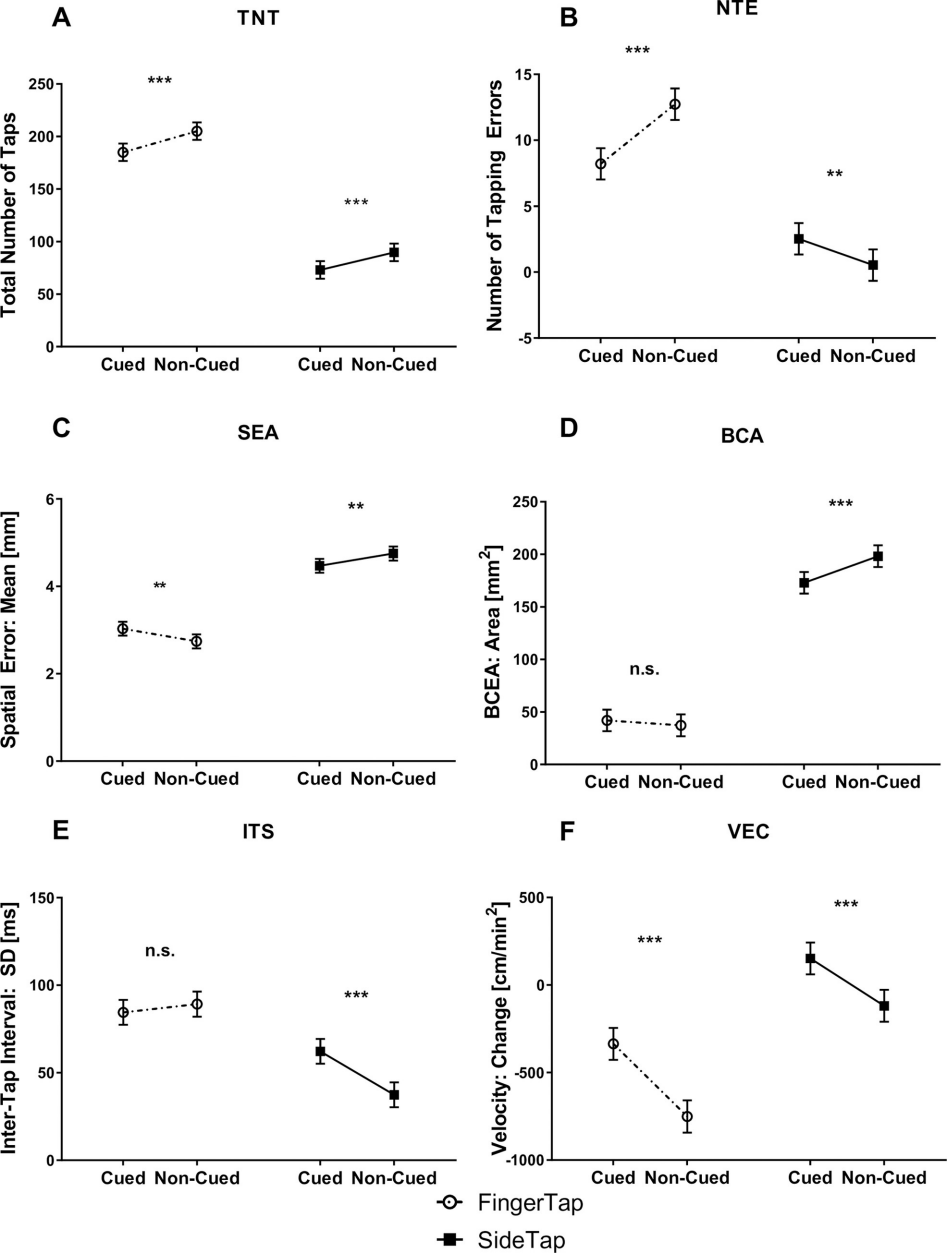
The effects of configuration and cueing on tapping performance. (A) TNT = Total Number of Taps (B) NTE = Number of Tapping Errors (C) SEA = Spatial Error: Mean (D) BCA = Bivariate Contour Ellipse Area (E) ITS = Inter Tap Interval: Standard Deviation (F) VEC = Velocity: Change. Values are based on estimated marginal means; error bars represent standard error of the marginal mean. * p < .05; **p < .01; ***p < .001; ns = not significant.

All estimated mean values for the tapping tasks, as well as all pairwise comparisons are presented in Table 8 and Fig 4. Participants tapped more often during AFT than AST, and during a non-cued versus a cued task. In addition, more tapping errors were made in AFT than AST. In the absence of the visual cue, participants made more tapping errors in the AFT task and fewer in the AST task. The mean spatial error was larger in AST than AFT. The non-cued task reduced and increased the mean spatial error in the AFT and AST configurations, respectively. The bivariate contour ellipse area was significantly larger in AST than AFT. The non-cued task increased the bivariate contour ellipse area only in the AST configuration. The SD of the inter-tap interval was lower in the AST configuration than in the AFT configuration. The absence of the visual cue reduced the SD of the inter-tap interval only in the AST configuration. The tapping velocity reduced throughout a measurement in both AFT tasks, with a steeper reduction in the non-cued tapping task. The tapping velocity increased throughout a measurement in cued AST, but reduced in the non-cued AST.

**Table 8:** Effect of task configuration and cueing on tapping performance.

		AFT		AST		Difference:	
Parameter		EMMean (SE)	ES	EMMean (SE)	ES	AFT–AST (SE)	ES
**TNT**	Cued	185.0 (8.31)		73.0 (8.30)		112 (4.25) ***	3.52
	Non-cued	205.1 (8.34)		89.70 (8.31)		115 (4.31) ***	3.62
	Diff (C—NC)	-20.01 (4.31) ***	-0.63	-16.70 (4.26) ***	-0.52		
**NTE**	Cued	8.21 (1.19)		2.52 (1.19)		5.7 (0.75) ***	1.02
	Non-cued	12.73 (1.20)		0.53 (1.19)		12.2 (0.76) ***	2.18
	Diff (C—NC)	-4.52 (0.76) ***	-0.81	1.99 (0.75) **	0.36	-	
**SEA**	Cued	3.03 (0.16)		4.47 (0.16)		-1.44 (0.1) ***	-1.93
	Non-cued	2.74 (0.16)		4.75 (0.16)		-2.01 (0.1) ***	-2.70
	Diff (C—NC)	0.29 (0.1) **	0.39	-0.28 (0.01) **	-0.37		
**BCA**	Cued	41.9 (10.3)		172.9 (10.3)		-131 (6.6) ***	-2.65
	Non-cued	37.2 (10.4)		198.2 (10.3)		-161 (6.7) ***	-3.25
	Diff (C—NC)	4.71 (6.7)	0.09	25.25 (6.6) ***	-0.51		
**ITS**	Cued	84.5 (7.10)		62.2 (7.09)		22.3 (5.81) ***	0.51
	Non-cued	89.2 (7.16)		37.4 (7.10)		51.9 (5.90) ***	1.19
	Diff (C—NC)	-4.77 (5.90)	-0.11	24.85 (5.81) ***	0.57		
**VEC**	Cued	-336 (91)		152 (90.9)		-488 (79.2) ***	-0.82
	Non-cued	-751 (91.9)		-119 (91)		-633 (40.4) ***	-1.07
	Diff (C—NC)	416 (91.0) ***	0.70	271 (79.2) ***	0.46		

Abbreviations: TNT = Total Number of Taps; NTE = Number of Tapping Errors; SEA = Spatial Error: Mean; BCA = Bivariate Contour Ellipse Area; ITS = Inter Tap Interval: Standard Deviation; VEC = Velocity: Change; EMMean = estimated marginal mean; ES = effect size, Cohen’s d; Diff = difference; C = cued; NC = Non-cued

* p < .05

** p < .01

*** p < .001

## Discussion

The current technical validation study provides several key contributions to the growing body of literature on touchscreen-based tapping devices. To the best of our knowledge, this study is the first to assess the effects of cueing and task configuration on tapping performance in a comparative manner. It is also the first study that explicitly assesses the repeatability and MDE of tapping parameters in healthy participants. Based on the results of the current study, recommendations for subsequent studies are discussed.

### Repeatability and minimal detectable effect

The first research question assessed the repeatability of tapping parameters across the four tapping tasks. Establishing good within-day repeatability is important as in clinical trials medication effects are often repeatedly assessed in a relatively short period of time [29]. Moreover, studies determining the acute pharmacodynamic effects of medication on a symptom, that may vary greatly between patients, (ideally) have a cross-over design. Hence, the optimal tapping task must provide repeatable parameters for the same subject both within and between testing visits. The within- and between-day repeatability were comparable for all reported parameters (see Tables 3 and 4). None of the parameters in any task showed significant changes between the four measurements within a day. This indicates the lack of significant learning effects when the measurements are repeated in a relatively short period of time. However, there was a significant effect of testing visit (the second visit occurred one week after the first) on the total number of taps, spatial error, and the standard deviation of the inter-tap interval. With the increase in number of taps at the second visit, the mean spatial error also increased. One explanation could be that as participants were already familiar with the task on the second visit, their priority might have shifted to speed rather than accuracy. To summarize, the within-day repeatability of the tapping parameters was good, but additional care should be taken when comparing repeated measures between testing visits.

The best repeatability was found in the speed related parameters, followed by accuracy, rhythm, and fatigue parameter. There were two parameters where lower repeatability was observed in AST compared to AFT, i.e., the number of tapping errors and the standard deviation of the inter-tap interval (i.e., rhythm parameter). The number of tapping errors showed lower repeatability values, especially in non-cued AST compared to the other tasks. Since most participants tapped correctly, there was little to no between-subject variation in tapping errors, lowering its ICC value. Additionally, the between-subject variance of the rhythm parameter was lower for AST compared to AFT. This finding suggests that it was easier for most people to tap with a steady rhythm during forearm muscle/ elbow joint driven motion than during AFT. Taken together, the AFT parameters generally resulted in better within-day repeatability than the AST ones, mainly driven by the increased between-subject variability in AFT.

The second research question assessed the parameters’ sensitivity to change in all four tapping tasks. Overall, the AST parameters were more sensitive compared to AFT parameters. The total number of taps showed moderate sensitivity in AFT and higher sensitivity in AST (i.e., MDE values ranging between 9.5%– 28%). Previous research indicates that the effect sizes observed on this parameter when comparing PD patients in an ON versus an OFF state, and when comparing PD patients with HCs, range within comparable boundaries [20, 21, 23, 25–27]. Although less frequently reported in literature, similar effect sizes were found in the mean spatial error and rhythm parameters [20, 25]. Given that PD patients tend to tap more arrhythmically [11, 14], slowly [20, 21, 28, 45], and less accurately [20, 28], the total number of taps, spatial error and the standard deviation of the inter-tap interval could be valuable parameters in subsequent clinical trials with patients.

### The effects of task configuration and cueing on tapping performance

In the AFT configuration, we found faster tapping, higher accuracy, worse rhythm, and more fatigue than in the AST configuration. The inter-target distance was 8 times smaller in AFT than AST, thereby reducing the travel time between two consecutive taps. AFT rhythm and fatigue effects, however, could primarily be explained by the increased muscle fatigue during fine, alternating finger movement as opposed to the upper-arm driven AST motion [25, 45, 46]. Why the increased speed was not associated with lower accuracy in AFT, could be explained by the position of the circles. The targets were placed under the natural position of the fingertips, making deviations from the center of the targets and tapping outside the target areas inherently less likely. Despite these two tasks being interchangeably used in the literature, researchers should be aware that AFT and AST are two different tasks, and they assess distinct motor functions.

Understanding the effects of cueing in finger tapping is crucial as cues can significantly improve motor performance in PD [34, 47]. In healthy participants, cueing reduced speed and fatigue for both AFT and AST, improved accuracy, and worsened rhythm for AST. In general, cueing had a larger effect on AST and seemed to be less relevant for AFT. The effects of cueing on tapping performance might be explained by the participant hesitating after each tap while waiting for the next circle to appear. More importantly, however, when participants tapped outside the target area, the next circle did not appear. Participants halted their hand movement, returned to correct the erroneous tap, resulting in higher inter-tap intervals, increased variability, lower fatigue, and fewer total taps. Hence cueing, rather than signaling the next target, provided immediate visual feedback. Considering a time-accuracy tradeoff, the immediate feedback and overall lower tapping speed can also account for the improved tapping accuracy in cued conditions. To summarize, cueing seemed to impair speed, rhythm, reduce fatigue, and improve accuracy of healthy participants, and it probably acted as visual feedback as opposed to a visual cue.

### Limitations and future research

The most important caveat of the current paper is that we did not assess a PD patient group. Hence, a natural continuation of this work would validate the AFT and AST against gold standard clinical scales in a patient population (i.e., the MDS-UPDRS). Whether PD patients perform better on AST compared to AFT, and whether AFT or AST is more sensitive to detect medication effects will be assessed in a currently ongoing clinical study. Moreover, the current study did not assess the pharmacological sensitivity of the task. The optimal tapping task(s) must also be able to detect medication changes, otherwise, the task(s)’ usefulness in clinical studies will be limited. In addition, even though we observed an increase in tapping speed on the second visit, we did not assess the exact nature of this effect. Future research should address the timescale and magnitude of testing visit effects on the tapping performance with respect to tapping style and/or motivation. Lastly, we did not vary the duration of the finger tapping tasks. Previous literature suggests that 30 seconds can be sufficient to detect fatigue effects [20], without overburdening the participants. Hence, the 30 second task length makes the set-up suitable for repeated testing, even when conducting studies with rapid-acting (dopaminergic) agents.

The findings, while preliminary, caution against the use of cueing in studies involving PD patients. Previous literature suggests that tapping speed, fatigue and rhythm are clinically relevant predictors of both PD related bradykinesia, as well as medication effects [11, 14, 48]. In healthy participants, cueing appears to impair the speed and rhythm of tapping, while reducing detectable fatigue. Hence, we argue that the tapping task set-up should be kept as simple as possible, to accurately detect potential differences in speed, rhythm, and change parameters, without inducing experimental noise. Additionally, exact comparisons with other studies remains difficult as technical specification on the implementation are not always reported (see Table 1). We encourage researchers to report on the technical implementation details of their tapping tasks (e.g., target distance, cueing, and duration).

Taken together, it seems preferable to use non-cued AST and AFT versions for further (validation) studies involving a PD population. The choice for AFT or AST should depend on the research question, as these tasks assess different aspects of movement. AFT appears to be more difficult for most healthy participants, and one could speculate that AFT would also be more difficult to perform for PD patients. For instance, Agostino [25, 45, 46] showed that it is significantly more difficult for PD patients to perform alternating finger tapping, as opposed to pronation-supination (i.e., forearm, elbow and shoulder driven movement), and Lalvay [25] showed that patients with severe parkinsonism have difficulties performing alternate finger tapping as opposed to one finger tapping. In addition, bradykinesia appears to worsen increasingly during isolated, sequential finger movements, as opposed to gross hand movements [45].

## Conclusion

The current study provides evidence that the custom developed AFT and AST tasks are well-functioning and repeatable measurement tools. From a technical point-of-view, they can be used in clinical trials assessing medication effects on bradykinesia. Recommended parameters are total number of taps, mean spatial error, and rhythm as they showed high repeatability and sensitivity. Moreover, the use of cueing in finger tapping tasks is unwarranted as visually cueing the tapping tasks can, in healthy participants, worsen tapping speed and rhythm, while improving accuracy. The choice for AFT or AST, should depend on the research question, as these tasks assess different aspects of movement. Concluding the technical validation step with encouraging results, the AFT and AST should be further investigated in subsequent studies with PD patients and in response to dopaminergic medication.

## Supporting information

S1 Appendix Experimental dataset. The experimental dataset can be found in S1 Appendix.
(CSV)
